# Potentially functional polymorphisms in *PAK1* are associated with risk of lung cancer in a Chinese population

**DOI:** 10.1002/cam4.524

**Published:** 2015-09-17

**Authors:** Mingfeng Zheng, Jia Liu, Meng Zhu, Rong Yin, Juncheng Dai, Jie Sun, Wei Shen, Yong Ji, Guangfu Jin, Hongxia Ma, Jing Dong, Lin Xu, Zhibin Hu, Hongbing Shen

**Affiliations:** 1 Department of Cardiothoracic Surgery, The Affiliated Wuxi People’s Hospital of Nanjing Medical UniversityWuxi, 214043, China; 2 Department of Epidemiology and Biostatistics, Collaborative Innovation Center of Cancer Medicine, School of Public Health, Nanjing Medical UniversityNanjing, 211166, China; 3 Jiangsu Key Lab of Cancer Biomarkers, Prevention and Treatment, Collaborative Innovation Center For Cancer Personalized Medicine, Nanjing Medical UniversityNanjing, 211166, China; 4 Jiangsu Key Laboratory of Molecular and Translational Cancer Research, Collaborative Innovation Center For Cancer Personalized Medicine, Nanjing Medical UniversityNanjing, 210009, China

**Keywords:** Interaction, lung cancer, *PAK1*, polymorphisms, susceptibility

## Abstract

P21-activated kinase 1(PAK1) plays an important role in the regulation of cell morphogenesis, motility, mitosis, and angiogenesis and has been implicated with tumorigenesis and tumor progression. We hypothesized that functional polymorphisms in *PAK1* gene may modify the risk of lung cancer. We screened four potentially functional polymorphisms (rs2154754, rs3015993, rs7109645, and rs2844337) in *PAK1* gene and evaluated the association between the genetic variants and lung cancer risk in a case**–**control study including 1341 lung cancer cases and 1982 cancer-free controls in a Chinese population. We found that variant allele of rs2154754 was significantly associated with a decreased risk of lung cancer (OR = 0.85, 95% CI: 0.77–0.95, *P *= 0.004), meanwhile the result of rs3015993 was marginal (OR = 0.90, 95%CI: 0.81–1.00, *P *= 0.044). After multiple comparisons, rs2154754 was still significantly associated with the lung cancer risk (*P *< 0.0125 for Bonferroni correction). We also detected a significant interaction between rs2154754 genotypes and smoking levels on lung cancer risk (*P *= 0.042). Combined analysis of these two polymorphisms showed a significant allele-dosage association between the number of protective alleles and reduced risk of lung cancer (*P*_trend_ = 0.008). These findings indicate that genetic variants in *PAK1* gene may contribute to susceptibility to lung cancer in the Chinese population.

## Introduction

Lung cancer is one of the most commonly diagnosed cancers and the leading cause of cancer deaths worldwide 1. Although lung cancer is mainly caused by tobacco use, genetic variations may determine individual outcome following exposure to tobacco carcinogens. Recently, genome-wide association studies (GWAS) have successfully identified dozens of loci that are associated with the risk of developing lung cancer 2–11. However, these loci can only interpret a small fraction of familiar risk of lung cancer 12. Because of the stringent screening criteria of GWAS, some potential causal variants and genes associated with lung cancer may be ignored. Thus, additional efforts on candidate genes that play important roles in lung carcinogenesis may be useful to uncover the missing heritability of lung cancer.

P21-activated kinase 1(PAK1) was initially identified as a downstream effector of the GTPases, Rac and Cdc42. Subsequent studies uncovered a variety of new functions for this kinase in growth factor and steroid receptor signaling, cytoskeleton remodeling, cell survival, oncogenic transformation, and gene transcription 13. In addition, *PAK1* was found to function in the regulation of cell morphogenesis, motility, mitosis, angiogenesis 14. Of interest, *PAK1* was frequently implicated in the tumorigenesis 15,16 and up-regulation of *PAK1* was frequently detected in various human cancers including lung cancer 16–19. Notably, PAK1 kinase can promote lung cancer cell motility, tumor migration, and invasion through activation of PAK1/LIMK1/cofilin pathway and CRK-II serine phosphorylation 20,21. These evidence collectively revealed the importance of *PAK1* in carcinogenesis, especially lung cancer.

Recently, two studies have demonstrated the associations between polymorphisms in *PAK1* gene and risk of papillary thyroid cancer and esophageal squamous cell carcinoma 22,23. However, the role of genetic variants in *PAK1* on the development of lung cancer is still unclear. In order to investigate whether genetic variants in *PAK1* contribute to susceptibility of developing lung cancer, we screened four potentially functional polymorphisms in *PAK1* gene and performed a genetic association analysis in a Chinese case**–**control study including 1341 lung cancer cases and 1982 cancer-free controls.

## Materials and Methods

### Study subjects

This case**–**control study was approved by the institutional review board of Nanjing Medical University. In total, 1341 lung cancer cases and 1982 controls were included. Patients with lung cancer were newly diagnosed and were consecutively recruited from the First Affiliated Hospital of Nanjing Medical University and the Cancer Hospital of Jiangsu Province since 2003. The histology for each case was histopathologically or cytologically confirmed by at least two local pathologists. Those who had a history of any cancer, metastasized cancer from other organs or had undergone radiotherapy or chemotherapy were excluded. Controls were selected from individuals participating screening of noncommunicable diseases in Jiangsu province. A total of 1982 controls were frequency-matched to lung cancer cases on age and gender. All of the subjects were genetically unrelated Han Chinese descent.

All participants signed the informed consent before taking part in this study. Each subject was face-to-face interviewed by trained interviewers to collect personal information on demographics data, cigarette smoking, and others. After the interview, approximately 5-mL venous blood was collected from each subject. Individuals were defined as smokers if they had smoked at an average of one cigarette or more per day and for at least 1 year in their lifetime; otherwise, subjects were considered as nonsmokers. Pack-years of smoking were defined as packs per day multiply smoking duration years. Smokers were divided into light and heavy smokers according to the threshold of 25 pack-years. The distribution of characteristics are summarized in Table S1. The age and gender between cases and controls were comparable. Compared to controls, cases had a higher rate of smoking (61.08% vs. 48.54%) and heavy smokers (42.28% vs. 24.07%). Among case group, 481 subjects (35.87%) were diagnosed with squamous cell carcinoma and the others were diagnosed with adenocarcinoma.

### Potentially functional polymorphisms selection

Based on the NCBI database and HapMap single nucleotide polymorphism (SNP) database, common SNPs (minor allele frequency, MAF ≥ 5%) in Chinese Han population were screened in gene regions (including 10-kb up-stream region of *PAK1*). After functional prediction by using SNPinfo Web Server (http://snpinfo.niehs.nih.gov/), a total of seven potentially functional SNPs were selected (Table S2). SNPs with low linkage disequilibrium analysis (*r*^2^ < 0.8) were retained. As a result, four functional SNPs (rs2154754, rs3015993, rs7109645, and rs2844337) were selected to be genotyped (Table S3).

### Genotyping

Genomic DNA was isolated from leukocyte pellets of venous blood by proteinase K digestion and followed by phenolchloroform extraction and ethanol precipitation. The genotyping was performed by using Illumina Infinium® BeadChip (Illumina Inc, San Diego, CA, USA) and the genotype calling was performed using the GenTrain version 1.0 clustering algorithm in GenomeStudio V2011.1 (Illumina). Technicians who undertook the genotyping assays were blinded to the subjects’ case or control status. Overall call rates of the 4 SNPs were from 99.64% to 100% (Table S3).

### Statistical analysis

The *χ*^2^ test for categorical variables and Student’s *t*-tests for continuous variables were used to analyze distribution differences of demographic characteristics and genotypes between cases and controls. Hardy**–**Weinberg equilibrium for the distribution of each SNP was evaluated using the goodness-of-fit χ^2^ test by comparing the observed genotype frequencies with the expected ones among the controls. Odd ratios (ORs) and their 95% confidence intervals (CI) were calculated by using logistic regression analyses to evaluate the association between SNPs and lung cancer risk with an adjustment for age, gender and pack-years of smoking. To examine the differences between subgroups, the *χ*^2^- based *Q*-test was used to test the heterogeneity of effect sizes (ORs and 95% CIs) derived from corresponding subgroups. Multiplicative interactions were tested using a general logistic regression model by applying the equation:

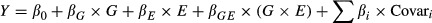
where *Y* is the logit of case-control status, *G* is SNP and *E* is environmental factor: pack year, _β0_ is constant, *β*_*G*_ and β_*E*_ are the main effects of factors *G* and *E*, respectively, *β*_*GE*_ is the interaction term, Covar_*i*_ are the covariates for adjustment, including age and gender. All of the statistical analyses were performed using R software (version 2.15.3; The R Foundation for Statistical Computing, http://www.cran.r-project.org/) and Stata Version 10.0 software (Stata, College Station, TX).

## Results

The observed genotype frequencies for the four genotyped SNPs were all in agreement with Hardy**–**Weinberg equilibrium in the controls (*P *> 0.05) (Table S3). Logistic regression analyses revealed that the A allele of rs2154754 was significantly associated with decreased risk of lung cancer (OR = 0.85, 95% CI: 0.77–0.95, *P = *0.004) (Table1). In addition, the T allele of rs3015993 was marginally associated with decreased risk of lung cancer (OR = 0.90, 95% CI: 0.81–1.00, *P *= 0.044). After multiple comparisons, rs2154754 was still significantly associated with the lung cancer risk (*P *< 0.0125 for Bonferroni correction).However, there was no obvious evidence for the associations between the remaining 2 SNPs (rs7109645 and rs2844337) and lung cancer risk.

**Table 1:** Associations between potentially functional SNPs in *PAK1* gene and lung cancer risk

Genotype	Case*N* (%)	Control*N* (%)	OR (95% CI)^2^	*P* 2
rs2154754(G>A)^1^				
GG	679 (50.63)	920 (46.42)	1	
GA	545 (40.64)	837 (42.23)	0.89 (0.77–1.04)	0.142
AA	117 (8.73)	225 (11.35)	0.69 (0.54–0.89)	0.004
Additive model			0.85 (0.77–0.95)	0.004
rs3015993(A>T)^1^				
AA	361 (26.92)	479 (24.17)	1	
AT	672 (50.11)	1011 (51.01)	0.87 (0.73–1.03)	0.106
TT	308 (22.97)	492 (24.82)	0.81 (0.66–1.00)	0.046
Additive model			0.90 (0.81–1.00)	0.044
rs7109645(A>C)^1^				
AA	842 (62.79)	1306 (65.89)	1	
AC	445 (33.18)	603 (30.42)	1.13 (0.96–1.31)	0.132
CC	54 (4.03)	73 (3.69)	1.08 (0.74–1.56)	0.700
Additive model			1.09 (0.96–1.24)	0.182
rs2844337(A>C)^1^				
AA	832 (62.28)	1274 (64.51)	1	
AC	453 (33.91)	629 (31.85)	1.08 (0.92–1.26)	0.345
CC	51 (3.81)	72 (3.64)	1.06 (0.73–1.55)	0.747
Additive model			1.06 (0.93–1.20)	0.373

1 Major allele > Minor allele.

2 Logistic regression with adjustment for age, gender, and pack-years of smoking.

Furthermore, in the stratification analysis, the association between rs2154754, rs3015993, and lung cancer risk were evaluated in subgroups based on age, gender, smoking status, pack-years of smoking, and histology type of lung cancer. As shown in Table2, there were no significant differences between subgroups for the association of the two SNPs with lung cancer risk. Interestingly, stronger effects of these two SNPs were observed among smokers (rs2154754: OR = 0.75, 95% CI: 0.64–0.88, *P *< 0.001; and rs3015993: OR = 0.80, 95% CI: 0.69–0.93, *P *= 0.004) and heavy smokers (rs2154754: OR = 0.79, 95% CI: 0.66–0.95, *P *= 0.011; and rs3015993: OR = 0.83, 95% CI: 0.70–0.99, *P *= 0.040).

**Table 2:** Stratified analysis on the associations of rs2154754 and rs3015993 in *PAK1* with lung cancer risk

Characteristics	rs2154754(G>A)				*P*_*het*_ 3	rs3015993(A>T)				*P*_*het*_ 3
	Case^1^	Control^1^	OR (95% CI)^2^	*P* 2		Case^1^	Control^1^	OR (95% CI)^2^	*P* 2	
Age										
≤60	307/241/48	404/377/102	0.80 (0.68–0.95)	0.008	0.342	176/296/124	213/444/226	0.81 (0.70–0.95)	0.008	0.067
>60	372/304/69	516/460/123	0.89 (0.77–1.03)	0.114		185/376/184	266/567/266	0.97 (0.85–1.12)	0.710	
Gender										
Male	507/358/84	621/588/149	0.79 (0.69–0.90)	0.001	0.076	257/479/213	321/698/339	0.87 (0.76–0.98)	0.022	0.387
Female	172/187/33	299/249/76	0.98 (0.81–1.20)	0.857		104/193/95	158/313/153	0.96 (0.80–1.16)	0.675	
Smoking status										
Current	341/241/52	399/376/101	0.75 (0.64–0.88)	<0.001	0.091	165/339/130	192/459/225	0.80 (0.69–0.93)	0.004	0.181
Former	94/72/19	46/31/9	1.03 (0.70–1.52)	0.881		50/84/51	22/44/20	1.02 (0.71–1.46)	0.917	
Never	244/232/46	475/430/115	0.94 (0.79–1.10)	0.425		146/249/127	265/508/247	0.96 (0.83–1.12)	0.630	
Pack-years										
0	244/232/46	475/430/115	0.94 (0.79–1.10)	0.425	0.321	146/249/127	265/508/247	0.96 (0.83–1.12)	0.630	0.404
0–25	129/104/19	226/204/55	0.80 (0.63–1.02)	0.066		64/136/52	108/261/116	0.84 (0.66–1.05)	0.129	
>25	306/209/52	219/203/55	0.79 (0.66–0.95)	0.011		151/287/129	106/242/129	0.83 (0.70–0.99)	0.040	
Histology type										
Squamous cell carcinoma	245/193/43	920/837/225	0.86 (0.73–1.01)	0.074	0.913	134/236/111	479/1011/492	0.88 (0.75–1.03)	0.109	0.737
Adenocarcinoma	434/352/74	920/837/225	0.85 (0.75–0.96)	0.009		227/436/197	479/1011/492	0.91 (0.81–1.03)	0.131	

1 Wild-type homozygote/heterozygote/variant homozygote.

2 Adjusted for age, gender, and pack-years of smoking where appropriate in additive model.

3 *P* for heterogeneity.

In order to investigate whether the effect of the identified variants on lung cancer was modified by cigarette smoking, we performed interaction analysis between the two SNPs and smoking levels. We identified a significant multiplicative interaction between rs2154754 and smoking levels on lung cancer risk (*P* for multiplicative interaction = 0.042) (Table3). However, no significant multiplicative interaction was observed between rs3015993 and pack-years of smoking on lung cancer risk (data not shown).

**Table 3:** The interaction between rs2154754 genotypes and smoking levels on lung cancer risk

Smoking levels	Genotype	Case*N* (%)	Control*N* (%)	OR (95% CI)	*P* 1
0	GA/AA	278 (20.73)	545 (27.50)	1	
0	GG	244 (18.20)	475 (23.97)	1.01 (0.81–1.24)	0.973
0–25	GA/AA	123 (9.17)	259 (13.07)	1.17 (0.88–1.57)	0.282
0–25	GG	129 (9.62)	226 (11.40)	1.42 (1.06–1.91)	0.019
>25	GA/AA	261 (19.46)	258 (13.02)	2.68 (2.06–3.47)	<0.001
>25	GG	306 (22.82)	219 (11.04)	3.71 (2.85–4.83)	<0.001
*P* for multiplicative interaction					0.042

1 Adjusting for age and gender.

We also conducted a combined analysis to evaluate the cumulative effect of the two identified SNPs. As expect, we found a significant allele-dosage association between number of protective alleles and lung cancer risk (*P*_trend_ = 0.008). As shown in Table4, compared with individuals without protective alleles, those carrying “4” protective alleles had a 33% (OR = 0.67, 95% CI: 0.51–0.87, *P *= 0.003) reduced risk of developing lung cancer. These data indicated that individuals carrying more protective alleles had a lower risk of lung cancer.

**Table 4:** Combined effects of rs2154754 and rs3015993 on lung cancer risk

Number of protective alleles	Case*N* (%)	Control*N* (%)	OR (95% CI)	*P* 1
0	361 (26.92)	479 (24.17)	1	
1	272 (20.28)	378 (19.07)	0.92 (0.74–1.14)	0.459
2	446 (33.26)	696 (35.12)	0.84 (0.70–1.01)	0.071
3	145 (10.81)	204 (10.29)	0.94 (0.72–1.22)	0.648
4	117 (8.73)	225 (11.35)	0.67 (0.51–0.87)	0.003
Trend				0.008

1 Derived from logistic regression with an adjustment for age, gender, and pack-years of smoking.

## Discussion

In our present study, we evaluated the association of four potentially functional polymorphisms in the *PAK1* with lung cancer risk in a case**–**control study including 1341 cases and 1982 controls in a Chinese population. Two SNPs (rs2154754 and rs3015993) were identified to be significantly associated with lung cancer risk. The SNP rs2154754 was detected with an approximately 15% decreased risk and rs3015993 was detected with 10% decreased risk of lung cancer risk in our population. The SNP rs2154754 kept significant after multiple comparisons. To the best of our knowledge, this is the first association study of polymorphisms in *PAK1* and lung cancer.

The *PAK1* gene is located on 11q13-q14 and this region is frequently amplified in many human cancers, with a complex structure harboring multiple potential oncogenic drivers 22,24. Emerging evidences have suggested that *PAK1* plays an important role in carcinogenesis and tumor progression 13,14. In special, for lung cancer *PAK1* could promote cell motility, tumor migration, and invasion through different mechanism 20,21. Our current study provided genetic evidence at the population level that *PAK1* may be involved in the carcinogenesis and development of lung cancer and further highlighted the important role of genetic variants in *PAK1* as molecular biomarkers for lung cancer susceptibility.

Before multiple comparisons, there were two positive SNPs (rs2154754 and rs3015993). The SNPs rs2154754 and rs3015993 are both located on 15th intron of *PAK1*. Recently, mutations in intron have been reported to lead to transcriptional dysregulation 25. According to the web-based SNP analysis tool: SNPinfo, the base change for rs2154754 and rs3015993 may influence the binding of the transcription factors and then lead to transcriptional dysregulation of *PAK1*.We then performed functional annotation for the two SNPs, as well as those tagged by the two SNPs (*r*^2^ > 0.8) based on DNase-seq database and RegulomeDB (http://regulome.stanford.edu/) (Table S4) 26. Among the two identified SNPs and those 77 highly correlated SNPs, seven SNPs are within open chromatin regions associated with gene regulatory elements. Furthermore, 10 SNPs located in motifs may influence the binding of specified transcription factors. These suggest that the 2 marker SNPs and those tagged by which may transcriptionally modulate the expression of *PAK1* gene. It is plausible that these variants may result in the aberrant activities of certain transcription factors. In turn, these factors interactively regulate the expression of the *PAK1* gene, hence regulating lung carcinogenesis through different mechanisms. However, no experimental evidence validates this hypothesis and future functional studies are warranted to clarify this point.

Notably, we found that the effect of rs2154754 mainly restricted on current smokers, especially on heavy smokers. The genotypes of rs2154754 showed significant interaction with smoking levels on lung cancer risk. Zhang et al. 27 reported that cigarette exposure could activate the phosphatidylinositol 3-kinase (PI3K)-Akt pathway and the proto-oncogene *FRA-1*, which is known to up-regulate the expression of genes involved in tumor progression and invasion. They also found that the PI3K through *PAK1* regulates *FRA-1* proto-oncogene induction by cigarette smoke and the subsequent activation of the Elk1 and cAMP-response element-binding protein transcription factors. This finding can partly interpret our interaction outcome. It is plausible that variant genotypes of rs2154754 may regulate the expression of the *PAK1* gene and then regulate the cigarette smoke-induced *FRA-1* proto-oncogene expression, subsequently altering the role of *FRA-1* on up-regulating the expression of genes involved in tumor progression and invasion. However, this hypothesis is very preliminary and merit further experimental investigation.

In summary, our study investigated the role of genetic variants in *PAK1* gene in lung cancer development in a Chinese population. Our results suggested, for the first time, that genetic variants in *PAK1* may modify the risk of lung cancer. Further independent studies incorporating functional evaluations are warranted to validate the association and clarify the biological mechanisms of these SNPs in lung cancer development.

## Supporting Information

**Table S1.** Distributions of selected variables in lung cancer cases and cancer-free controls. **Table S2.** Functional annotation of seven functional single nucleotide polymorphism (SNPs) in *PAK1* gene according to SNPinfo. **Table S3.** Summary of four functional single nucleotide polymorphisms in *PAK1* gene. **Table S4.** Functional annotation for the two marker single nucleotide polymorphism (SNPs) and those with strong linkage disequilibrium with the two marker SNPs.

## References

[b1] Ferlay J, Soerjomataram I, Dikshit R, Eser S, Mathers C, Rebelo M (2015). Cancer incidence and mortality worldwide: sources, methods and major patterns in GLOBOCAN 2012. Int. J. Cancer.

[b2] Hung RJ, McKay JD, Gaborieau V, Boffetta P, Hashibe M, Zaridze D (2008). A susceptibility locus for lung cancer maps to nicotinic acetylcholine receptor subunit genes on 15q25. Nature.

[b3] Amos CI, Wu X, Broderick P, Gorlov IP, Gu J, Eisen T (2008). Genome-wide association scan of tag SNPs identifies a susceptibility locus for lung cancer at 15q25.1. Nat. Genet.

[b4] Liu P, Vikis HG, Wang D, Lu Y, Wang Y, Schwartz AG (2008). Familial aggregation of common sequence variants on 15q24-25.1 in lung cancer. J. Natl. Cancer Inst.

[b5] Wang Y, Broderick P, Webb E, Wu X, Vijayakrishnan J, Matakidou A (2008). Common 5p15.33 and 6p21.33 variants influence lung cancer risk. Nat. Genet.

[b6] McKay JD, Hung RJ, Gaborieau V, Boffetta P, Chabrier A, Byrnes G (2008). Lung cancer susceptibility locus at 5p15.33. Nat. Genet.

[b7] Broderick P, Wang Y, Vijayakrishnan J, Matakidou A, Spitz MR, Eisen T (2009). Deciphering the impact of common genetic variation on lung cancer risk: a genome-wide association study. Cancer Res.

[b8] Hu Z, Wu C, Shi Y, Guo H, Zhao X, Yin Z (2011). A genome-wide association study identifies two new lung cancer susceptibility loci at 13q12.12 and 22q12.2 in Han Chinese. Nat. Genet.

[b9] Shiraishi K, Kunitoh H, Daigo Y, Takahashi A, Goto K, Sakamoto H (2012). A genome-wide association study identifies two new susceptibility loci for lung adenocarcinoma in the Japanese population. Nat. Genet.

[b10] Dong J, Hu Z, Wu C, Guo H, Zhou B, Lv J (2012). Association analyses identify multiple new lung cancer susceptibility loci and their interactions with smoking in the Chinese population. Nat. Genet.

[b11] Lan Q, Hsiung CA, Matsuo K, Hong YC, Seow A, Wang Z (2012). Genome-wide association analysis identifies new lung cancer susceptibility loci in never-smoking women in Asia. Nat. Genet.

[b12] Manolio TA, Collins FS, Cox NJ, Goldstein DB, Hindorff LA, Hunter DJ (2009). Finding the missing heritability of complex diseases. Nature.

[b13] Eswaran J, Li DQ, Shah A, Kumar R (2012). Molecular pathways: targeting p21-activated kinase 1 signaling in cancer–opportunities, challenges, and limitations. Clin. Cancer Res.

[b14] Qing H, Gong W, Che Y, Wang X, Peng L, Liang Y (2012). PAK1-dependent MAPK pathway activation is required for colorectal cancer cell proliferation. Tumour Biol.

[b15] Hammer A, Rider L, Oladimeji P, Cook L, Li Q, Mattingly RR (2013). Tyrosyl phosphorylated PAK1 regulates breast cancer cell motility in response to prolactin through filamin A. Mol. Endocrinol.

[b16] Zhou W, Jubb AM, Lyle K, Xiao Q, Ong CC, Desai R (2014). PAK1 mediates pancreatic cancer cell migration and resistance to MET inhibition. J. Pathol.

[b17] Ong CC, Jubb AM, Haverty PM, Zhou W, Tran V, Truong T (2011). Targeting p21-activated kinase 1 (PAK1) to induce apoptosis of tumor cells. Proc. Natl. Acad. Sci. USA.

[b18] Rider L, Oladimeji P, Diakonova M (2013). PAK1 regulates breast cancer cell invasion through secretion of matrix metalloproteinases in response to prolactin and three-dimensional collagen IV. Mol. Endocrinol.

[b19] Kim E, Youn H, Kwon T, Son B, Kang J, Yang HJ (2014). PAK1 tyrosine phosphorylation is required to induce epithelial-mesenchymal transition and radioresistance in lung cancer cells. Cancer Res.

[b20] Jang I, Jeon BT, Jeong EA, Kim EJ, Kang D, Lee JS (2012). Pak1/LIMK1/Cofilin Pathway Contributes to Tumor Migration and Invasion in Human Non-Small Cell Lung Carcinomas and Cell Lines. Korean J. Physiol. Pharmacol.

[b21] Rettig M, Trinidad K, Pezeshkpour G, Frost P, Sharma S, Moatamed F (2012). PAK1 kinase promotes cell motility and invasiveness through CRK-II serine phosphorylation in non-small cell lung cancer cells. PLoS ONE.

[b22] Hidalgo M, Saez ME, Martinez-Tello FJ, Moron FJ, Ferrero-Herrero E, Labalde-Martinez M (2008). Absence of allelic imbalance involving EMSY, CAPN5, and PAK1 genes in papillary thyroid carcinoma. J. Endocrinol. Invest.

[b23] Chattopadhyay I, Singh A, Phukan R, Purkayastha J, Kataki A, Mahanta J (2010). Genome-wide analysis of chromosomal alterations in patients with esophageal squamous cell carcinoma exposed to tobacco and betel quid from high-risk area in India. Mutat. Res.

[b24] Wilkerson PM, Dedes KJ, Wetterskog D, Mackay A, Lambros MB, Mansour M (2011). Functional characterization of EMSY gene amplification in human cancers. J. Pathol.

[b25] Yu Q, Shen XH, Li Y, Li RJ, Li J, Luo YY (2013). An intron mutation in the ACVRL1 may be associated with a transcriptional regulation defect in a Chinese family with hereditary hemorrhagic telangiectasia. PLoS ONE.

[b26] Chu M, Zhang R, Zhao Y, Wu C, Guo H, Zhou B (2014). A genome-wide gene-gene interaction analysis identifies an epistatic gene pair for lung cancer susceptibility in Han Chinese. Carcinogenesis.

[b27] Zhang Q, Adiseshaiah P, Kalvakolanu DV, Reddy SP (2006). A Phosphatidylinositol 3-kinase-regulated Akt-independent signaling promotes cigarette smoke-induced FRA-1 expression. J. Biol. Chem.

