# Supplementary material for: Potentially functional polymorphisms in PAK1 are associated with risk of lung cancer in a Chinese population
Source: Cancer Med. 2015 Sep 17;4(11):1781–7. doi: 10.1002/cam4.524 (PMC4674004; doi:10.1002/cam4.524)
Supplement: Supplementary file 1 [file cam40004-1781-sd1.docx]

**Supplementary Table 1 . Distributions of selected variables in lung cancer cases and cancer-free controls**

| **Variables** | **Case (N=1,341)** | **Control（N=1,982）** | ***P*** |
| --- | --- | --- | --- |
| Age(mean±sd) | 61.06±10.15 | 61.32±11.07 | 0.473 |
| ≤60 | 596 (44.44%) | 883(44.55%) | 0.980 |
| >60 | 745(55.56%) | 1099(55.45%) |  |
| Gender |  |  |  |
| Male | 949(70.77%) | 1358(68.52%) | 0.179 |
| Female | 392(29.23%) | 624(31.48%) |  |
| Smoking status |  |  |  |
| Current | 634(47.28%) | 876(44.20%) | <0.001 |
| Former | 185(13.80%) | 86(4.34%) |  |
| Never | 522(38.92%) | 1020(51.46%) |  |
| Pack-year(py) |  |  |  |
| ≤25 | 774(57.72%) | 1505(75.93%) | <0.001 |
| >25 | 567(42.28%) | 477(24.07%) |  |
| Histology type |  |  |  |
| Squamous cell carcinoma | 481(35.87%) |  |  |
| Adenocarcinoma | 860(64.13%) |  |  |

**Supplementary Table 2. Functional annotation of 7 functional SNPs in *PAK1* gene according to SNPinfo**

| SNP | *r^2^* | TFBS^a^ | Splicing(site) | Splicing(ESE or ESS)^b^ | Splicing(abolish domain) | miRNA(miRanda) | miRNA(Sanger) | nsSNP^c^ | Stop Codon |
| --- | --- | --- | --- | --- | --- | --- | --- | --- | --- |
| rs2154754 | 1 | Y | -- | -- | -- | -- | -- | -- | -- |
| rs11237200 |  | Y | -- | -- | -- | -- | -- | -- | -- |
| rs3015993 | 0.837 | Y | -- | -- | -- | -- | -- | -- | -- |
| rs2729762 |  | -- | -- | -- | -- | Y | -- | -- | -- |
| rs2844337 |  | -- | -- | -- | -- | Y | -- | -- | -- |
| rs7109645 | 0.895 | Y | -- | -- | -- | -- | -- | -- | -- |
| rs7943778 |  | Y | -- | -- | -- | -- | -- | -- | -- |

^a^ Transcription factor binding sites;

^b^ ESE: Exonic splicing enhancer; ESS: Exonic splicing silencer;

^c^ Non-synonymous coding SNPs.

**Supplementary Table 3. Summary of 4 functional SNPs in *PAK1* gene**

| **SNP** | **Base change ^a^** | **Location** | **Genotyping Rate (%)** | **MAF^b^ (case/control)** | **HWE^c^** |
| --- | --- | --- | --- | --- | --- |
| rs2154754 | G>A | intron | 100 | 0.29/0.32 | 0.101 |
| rs3015993 | A>T | intron | 100 | 0.48/0.50 | 0.393 |
| rs7109645 | A>C | 5' near gene | 100 | 0.21/0.19 | 0.769 |
| rs2844337 | A>C | 3' UTR | 99.64 | 0.21/0.20 | 0.668 |

^a^ Major allele > Minor allele;

^b^ Minor allele frequency;

^c^ Hardy–Weinberg equilibrium test among controls.

**Supplementary Table 4. Functional annotation for the 2 marker SNPs and those with strong linkage disequilibrium with the 2 marker SNPs**

| SNP | Marker SNP | *r*^2^ | Open chromatin | Regulome DB Score ^a^ | Hits | Protein binding | |
| --- | --- | --- | --- | --- | --- | --- | --- |
| rs3019249 | rs2154754 | 1 |  | 2c | Motifs, Chromatin structure  Histone modification | GATA6, GATA1 | |
| rs503406 | rs2154754 | 1 | open chromatin | 4 | Chromatin structure  Histone modification | GATA6 | |
| rs11237200 | rs2154754 | 1 | open chromatin | 4 | Chromatin structure  Histone modification | HNF4A | |
| rs595654 | rs2154754 | 1 |  | 5 | Histone modification | GATA1 | |
| rs531638 | rs2154754 | 1 | open chromatin | 5 | Chromatin structure  Histone modification |  | |
| **rs2154754** | rs2154754 |  |  | 6 | Motifs, Histone modification |  | |
| rs476258 | rs2154754 | 0.94 |  | 6 | Motifs, Histone modification |  | |
| rs4945161 | rs2154754 | 0.90 |  | 6 | Motifs, Histone modification |  | |
| rs1670445 | rs2154754 | 0.86 |  | 6 | Motifs, Histone modification |  | |
| rs505841 | rs2154754 | 1 |  | 7 | No data |  | |
| rs1237490 | rs2154754 | 1 |  | 7 | No data |  | |
| rs495889 | rs3015993 | 1 |  | 2b | Motifs, Chromatin structure  Histone modification | E2F1,ZNF263,AR,CDX2,  HNF4A,GATA2,E2F4 | |
| rs3015984 | rs3015993 | 0.91 | open chromatin | 4 | Chromatin structure  Histone modification | EP300,GATA2 | |
| rs575662 | rs3015993 | 0.89 |  | 4 | Chromatin structure  Histone modification | HNF4A | |
| rs674652 | rs3015993 | 1 |  | 5 | Chromatin structure  Histone modification | GATA6 | |
| SNP | Marker SNP | *r*^2^ | Open chromatin | Regulome DB Score ^a^ | Hits | Protein binding | |
| rs499880 | rs3015993 | 1 |  | 5 | Motifs, Chromatin structure  Histone modification |  | |
| rs568309 | rs3015993 | 1 | open chromatin | 5 | Chromatin structure  Histone modification |  | |
| rs688212 | rs3015993 | 0.98 | open chromatin | 5 | Motifs, Chromatin structure  Histone modification |  | |
| rs628325 | rs3015993 | 0.93 |  | 5 | Histone modification | GATA1,TAL1 | |
| rs683497 | rs3015993 | 0.93 |  | 5 | Chromatin structure  Histone modification |  | |
| rs539906 | rs3015993 | 0.91 |  | 5 | Chromatin structure  Histone modification |  | |
| rs3019240 | rs3015993 | 0.91 |  | 5 | Motifs, Chromatin structure  Histone modification |  | |
| rs482243 | rs3015993 | 0.89 |  | 5 | Motifs, Chromatin structure  Histone modification |  | |
| rs2729762 | rs3015993 | 0.84 | open chromatin | 5 | Chromatin structure  Histone modification |  | |
| rs57129983 | rs3015993 | 0.83 |  | 5 | Motifs, Histone modification | SETDB1 | |
| rs2007466 | rs3015993 | 0.83 |  | 5 | Chromatin structure  Histone modification |  | |
| rs3015991 | rs3015993 | 1 |  | 6 | Motifs, Histone modification |  | |
| rs556696 | rs3015993 | 0.98 |  | 6 | Motifs, Histone modification |  | |
| rs492527 | rs3015993 | 0.98 |  | 6 | Motifs, Histone modification |  | |
| rs98703 | rs3015993 | 0.98 |  | 6 | Motifs, Histone modification |  | |
| rs576997 | rs3015993 | 0.93 |  | 6 | Motifs, Histone modification |  | |
| SNP | Marker SNP | *r*^2^ | Open chromatin | Regulome DB Score ^a^ | Hits | Protein binding | |
| rs614775 | rs3015993 | 0.93 |  | 6 | Motifs, Histone modification |  | |
| rs605910 | rs3015993 | 0.91 |  | 6 | Motifs, Histone modification |  | |
| rs491781 | rs3015993 | 0.91 |  | 6 | Motifs, Histone modification |  | |
| rs73496890 | rs3015993 | 0.91 |  | 6 | Motifs, Histone modification |  | |
| rs3019243 | rs3015993 | 0.91 |  | 6 | Motifs, Histone modification |  | |
| rs538670 | rs3015993 | 0.91 |  | 6 | Motifs, Histone modification |  | |
| rs489038 | rs3015993 | 0.89 |  | 6 | Motifs, Histone modification |  | |
| rs576079 | rs3015993 | 0.89 |  | 6 | Motifs, Histone modification |  | |
| rs497172 | rs3015993 | 0.89 |  | 6 | Motifs, Histone modification |  | |
| rs497946 | rs3015993 | 0.89 |  | 6 | Motifs, Histone modification |  | |
| rs2725812 | rs3015993 | 0.89 |  | 6 | Motifs, Chromatin structure  Histone modification |  | |
| rs4945169 | rs3015993 | 0.87 |  | 6 | Motifs, Histone modification |  | |
| rs1225479 | rs3015993 | 0.85 |  | 6 | Motifs, Histone modification |  | |
| rs34420077 | rs3015993 | 0.83 |  | 6 | Motifs, Histone modification |  | |
| rs76983095 | rs3015993 | 0.81 |  | 6 | Motifs, Histone modification |  | |
| rs11824060 | rs3015993 | 0.81 |  | 6 | Motifs, Histone modification |  | |
| rs4945162 | rs3015993 | 0.81 |  | 6 | Motifs, Histone modification |  | |
| **rs3015993** | rs3015993 |  |  | 7 | No data |  | |
| SNP | Marker SNP | *r*^2^ | Open chromatin | Regulome DB Score ^a^ | Hits | Protein binding | |
| rs3015989 | rs3015993 | 1 |  | 7 | No data |  | |
| rs478475 | rs3015993 | 1 |  | 7 | No data |  | |
| rs500731 | rs3015993 | 1 |  | 7 | No data |  | |
| rs635291 | rs3015993 | 0.98 |  | 7 | No data |  | |
| rs517511 | rs3015993 | 0.98 |  | 7 | No data |  | |
| rs476925 | rs3015993 | 0.98 |  | 7 | No data |  | |
| rs2853097 | rs3015993 | 0.98 |  | 7 | No data |  | |
| rs639857 | rs3015993 | 0.98 |  | 7 | No data |  | |
| rs473012 | rs3015993 | 0.95 |  | 7 | No data |  | |
| rs721382 | rs3015993 | 0.95 |  | 7 | No data |  | |
| rs10793248 | rs3015993 | 0.93 |  | 7 | No data |  | |
| rs3019258 | rs3015993 | 0.93 |  | 7 | No data |  | |
| rs584909 | rs3015993 | 0.91 |  | 7 | No data |  | |
| rs487322 | rs3015993 | 0.91 |  | 7 | No data |  | |
| rs3133294 | rs3015993 | 0.91 |  | 7 | No data |  | |
| rs561153 | rs3015993 | 0.91 |  | 7 | No data |  | |
| rs618012 | rs3015993 | 0.91 |  | 7 | No data |  | |
| rs478134 | rs3015993 | 0.91 |  | 7 | No data |  | |
| SNP | Marker SNP | *r*^2^ | Open chromatin | Regulome DB Score ^a^ | Hits | Protein binding | |
| rs562939 | rs3015993 | 0.91 |  | 7 | No data |  | |
| rs667331 | rs3015993 | 0.89 |  | 7 | No data |  | |
| rs599437 | rs3015993 | 0.89 |  | 7 | No data |  | |
| rs514191 | rs3015993 | 0.89 |  | 7 | No data |  | |
| rs3018485 | rs3015993 | 0.87 |  | 7 | No data |  | |
| rs4944158 | rs3015993 | 0.87 |  | 7 | No data |  | |
| rs4944153 | rs3015993 | 0.83 |  | 7 | No data |  | |
| rs7928731 | rs3015993 | 0.83 |  | 7 | No data |  | |
| rs61901802 | rs3015993 | 0.82 |  | 7 | No data |  | |
| rs11600983 | rs3015993 | 0.81 |  | 7 | No data |  | |
| rs4433591 | rs3015993 | 0.81 |  | 7 | No data |  | |
| rs4944155 | rs3015993 | 0.81 |  | 7 | No data |  | |
| ^a^ Description of Regulome DB :2b, TF binding + any motif +DNase footprint + DNase peak; 2c, TF binding + matched motif + DNase peak; 4, TF binding + DNase peak; 5, TF binding or DNase peak; 6, Motif hit; 7, No data supporting. | | | | | | |  |
